# Cultural Adaptation of a Web-Based Cognitive Behavioral Stress and Self-Management Intervention for Hispanic Sexual Minority Men Living with HIV and Cancer: A Mixed-Methods Feasibility Study

**DOI:** 10.1007/s10461-025-04945-y

**Published:** 2025-10-28

**Authors:** Blanca Noriega Esquives, Vanina Pavia, Marta Salazar, Marc Puccinelli, Frank J. Penedo, Sara St. George

**Affiliations:** 1 Department of Public Health Sciences, University of Miami Miller School of Medicine, 1120 NW 14th Street, Miami, FL 33136 USA; 2 Department of Psychology, University of Miami, Coral Gables, FL USA; 3 Department of Medicine, University of Miami Miller School of Medicine, Miami, FL USA

**Keywords:** Cancer, Cognitive behavioral stress and self-management, HIV, Hispanic, Intervention

## Abstract

This study aimed to describe the cultural adaptation and evaluation of a web-based cognitive behavioral stress and self-management (CBSM) intervention for Hispanic sexual minority men living with both HIV and cancer. Using a mixed-method single-group, pre-post design, we assessed the intervention’s feasibility, acceptability, and intended effects on health-related quality of life (HRQOL) and psychological distress. Fifteen Hispanic men participated in the study. Baseline assessments revealed low HRQOL, high levels of stress, and cancer-related PTSD symptoms. On average, participants attended seven of 10 sessions, with a 100% completion rate for pre- and post-intervention assessments. Most found the program relevant to their health needs, easy to navigate, and would recommend it. Participants demonstrated significant improvements in emotional well-being and on the PTSD avoidance subscale. Our findings emphasize the acceptability and feasibility of CBSM interventions for this population. However, recruitment challenges highlight the necessity for more inclusive and flexible strategies in future research.

**Supplementary Information:**

The online version contains supplementary material available at 10.1007/s10461-025-04945-y.

## Resumen

Este estudio tuvo como objetivo describir la adaptación cultural y evaluación de una intervención cognitivo-conductual para el manejo del estrés y autocuidado (CBSM, por sus siglas en inglés) ofrecida en línea para hombres hispanos de minorías sexuales que viven con VIH y cáncer. Usando un diseño de método mixto, de un solo grupo y pre-post, evaluamos su viabilidad, aceptabilidad y los efectos preliminares en la calidad de vida relacionada con la salud y el sufrimiento psicológico. Quince hispanos participaron en el estudio. Las evaluaciones iniciales revelaron baja calidad de vida, altos niveles de estrés y síntomas de trastorno de estrés postraumático (TEPT) relacionados con el cáncer. En promedio, los participantes asistieron a siete de las diez sesiones, completando el 100% de las evaluaciones anteriores y posteriores a la intervención. La mayoría consideró el programa relevante para sus necesidades de salud, fácil de navegar y lo recomendarían. Los participantes demostraron mejoras significativas en el bienestar emocional y en la subescala de evasión TEPT. Nuestros resultados enfatizan la aceptabilidad y viabilidad de intervenciones CBSM para esta población. Sin embargo, los desafíos de reclutamiento resaltan la necesidad de estrategias más inclusivas y flexibles en futuras investigaciones.

## Introduction

Hispanics in the U.S. are disproportionately affected by the HIV epidemic. In 2022, the HIV incidence rate for Hispanics was 4.4 times that of non-Hispanic Whites [1]. Additionally, Hispanic men had an HIV incidence rate eight times higher than that of Hispanic women [1]. Among men, sexual minority men (SMM) are the population most affected by HIV in the U.S. While non-Hispanic Black and White SMM experienced significant reductions in HIV incidence compared to 2018 estimates (16% and 20%, respectively), the HIV incidence among Hispanic SMM remained stable [1]. Furthermore, with the substantial increase in life expectancy for people living with HIV, more individuals are being diagnosed with chronic diseases, including cancer [2–4]. Cancer risk in people living with HIV results from a combination of traditional risk factors (e.g., age, obesity, and smoking), oncogenic co-infections (e.g., human papillomavirus and hepatitis C), and the prevalence of AIDS-defining cancers in people with uncontrolled HIV (e.g., Kaposi sarcoma and non-Hodgkin lymphoma) [5]. Importantly, people diagnosed with both HIV and cancer have poorer cancer outcomes, including higher rates of cancer recurrence and increased cancer-specific mortality, compared to those without HIV [5]. Recent studies have found that SMM are more likely to be diagnosed with cancer and report poorer HRQOL and cancer outcomes than heterosexual men [6–9]. Although estimates of HIV and cancer among sexual minorities by race/ethnicity are limited, Hispanic SMM are likely to constitute a substantial proportion of individuals living with HIV and cancer.

Individuals living with HIV and cancer may face multiple psychosocial and physical challenges as both conditions require complex, long-term and physically demanding treatments (e.g., antiretroviral therapy and chemotherapy), which are associated with higher risk of toxicity and harmful side effects. These treatments and the diseases themselves can lead to significant physical changes (e.g., weight loss, physical function decline, and changes in appearance), impacting patient’s self-esteem and quality of life [10]. Both diseases can also lead to substantial financial and practical burdens, such as high medical costs, time off from work, and securing transportation to appointments [11, 12]. A persistent sense of uncertainty is another significant stressor, as patients must manage the constant fear of cancer recurrence and potential HIV treatment failure [11].

Each disease also has its own unique set of stressors. Unlike many cancers that can be cured, HIV is a chronic, life-long condition requiring daily medication adherence, which can be difficult for those with unstable living conditions or limited resources [13]. HIV is also burdened with the stigma of being a sexually transmitted disease, leading to fear of disclosure, social rejection and isolation [14]. In contrast, cancer treatment, particularly those with curative intent such as chemotherapy and surgery, often involves intense and often debilitating regimens. Both patient populations share many coping strategies, including information seeking, where patients cope by educating themselves about their diagnosis, prognosis, and treatment options to feel more in control; social support, where patients lean on family and friends for emotional and practical support; and positive outlook, where patients often employ optimism and hope for finding meaning in their illness and managing fear [11, 15, 16].

The psychosocial difficulties linked to a dual diagnosis of HIV and cancer are likely exacerbated for ethnic and sexual minorities, who face disparities in HIV and cancer outcomes. Hispanic sexual minority men (SMM) with HIV and cancer might endure multiple stressors associated with their dual minority status, including limited access to care, fear of disclosure of their sexual identity to healthcare providers, chronic discrimination, stigmatization, and low satisfaction with medical services [11, 17–19]. These disparities underscore the urgent need for targeted interventions and support for Hispanics living with HIV and cancer, particularly Hispanic SMM, to decrease HIV incidence and enhance health outcomes.

Psychosocial interventions specifically tailored to the unique challenges of people living with both HIV and cancer are limited [20, 21]. Cognitive-behavioral stress and self-management (CBSM) seems well-suited for this population due to its emphasis on managing stress, enhancing coping skills, and alleviating symptom burden [22]. For example, the combined use of cognitive-behavioral stress techniques may help individuals manage the high stress, stigma, and depressive symptoms associated with a dual diagnosis, while the self-management component may equip them with skills for adhering to complex, lifelong HIV and cancer treatment regimens. CBSM is a group-based intervention delivered through the SmartManage web-based platform, consisting of ten weekly 90-minute sessions. Trained therapists (e.g., master’s level or psychology doctoral students) lead groups of 4–6 participants via videoconference, teaching cognitive-behavioral stress and self-management skills and relaxation techniques. The platform also offers additional resources, such as educational materials, relaxation exercises, and links to social support groups. Previous studies have demonstrated the efficacy of CBSM in reducing symptom burden and improving HRQOL among men with prostate cancer and women with breast cancer [23, 24]. Furthermore, CBSM has shown promising effects in SMM living with HIV, including reduced distress and depressive symptoms [25, 26]. While earlier research indicates that cultural factors significantly influence health outcomes among Hispanics, no interventions have been specifically designed for Hispanic SMM living with both HIV and cancer.

To address this gap, the purpose of this study was to (1) describe the cultural adaptation of a web-based CBSM intervention for Hispanic SMM living with HIV and cancer, (2) examine the feasibility and acceptability of the culturally tailored web-based CBSM intervention among dually diagnosed Hispanic SMM, and (3) determine the intended effects of the culturally tailored web-based CBSM intervention on health-related quality of life, general stress, and disease-related distress in Hispanic SMM living with HIV and cancer.

## Methods

### Cultural Adaptation of the CBSM Intervention

Our cultural adaptation process was guided by the heuristic framework developed by Barrera and Castro [27, 28]. This framework, previously utilized to adapt CBSM for Hispanic prostate cancer survivors [29], includes four stages of intervention adaptation: (1) information gathering, (2) preliminary adaptation design, (3) preliminary adaptation tests, and (4) adaptation refinement. This approach requires integrating both quantitative and qualitative data to make informed decisions about intervention adaptations, ensuring the content is culturally relevant. This study will report on the first three phases of the heuristic framework.

#### Stage 1: Information Gathering

Puccinelli and colleagues adapted the standard CBSM intervention to specifically address the complex needs of SMM living with both HIV and cancer [30]. This tailored version of CBSM included topics such as navigating the stigma associated with HIV and cancer, recognizing and managing chronic minority stress, coping with the dual burden of these chronic illnesses, optimizing medication and treatment adherence for both conditions, addressing sexual health concerns within the context of HIV and cancer treatments, and providing resources for accessing medical information and support. To further adapt this intervention for Hispanic SMM living with both HIV and cancer, we performed a literature search to gain a deeper understanding of the Hispanic SMM population, the challenges faced by Hispanic SMM living with HIV, and the impact of Hispanic cultural values, traditions, and illness perceptions on health. Key areas of focus included understanding how Hispanic cultural values influence health behaviors and coping mechanisms, identifying unique stressors experienced by Hispanic SMM, and exploring the role of family and community support in addressing the challenges faced by this population.

#### Stage 2: Preliminary Intervention Design

The next stage of the adaptation process involved making preliminary changes to the intervention based on our literature search. The culturally adapted CBSM intervention for Hispanic SMM living with HIV and cancer incorporated Hispanic cultural values such as machismo, fatalism, health stigma, likability/respect, familism, allocentrism, and external locus of control. Table 1 provides a detailed summary of the session-by-session content and its corresponding cultural adaptations. These modifications are reflected in both the intervention manual and the associated SmartManage platform.

**Table 1:** Hispanic cultural values and implications for CBSM

SmartManage sessions	CBSM content	Cultural adaptations
1) Introduction: HIV and Cancer Co-Management	Chronic minority stress Strategies for effective co-management of HIV and cancer, including medication adherence Self-advocacy Goal setting	Unique stressors experienced by Hispanic SMM Introducing Hispanic cultural values and their impact on health (i.e., Familism, Machismo, Likeability/Respect, Disease Stigma, Fatalism, external locus of control, allocentrism)
2) Stress and Stress Management	Defining stress Stress causes and consequences Introduction to mindfulness	Examples of stress specific to Hispanics (e.g., immigration, discrimination, acculturation, socioeconomic burden, social role strain). How Hispanic cultural values may aggravate stress
3) Linking Thoughts and Emotions	Introduction to appraisal process	Examples of cognitive processes or perceptions common in Hispanics (e.g., Fatalism – Thinking in black and white; Machismo – making statements with “I should…,”). How Hispanic cultural values can influence thoughts
4) Linking Thoughts and Emotions – Part 2	Cognitive distortions Negative thoughts and behaviors	
5) Sex and Intimacy	Creating a safe space Disclosure Addressing changes in roles after treatment Preventing HIV transmission	How Hispanic cultural values affect communication with others about sex (e.g., Respect/Likeability: Some men may not discuss sex with their doctors, as this may be seen as inappropriate or disrespectful.)
6) Effective communication	Managing emotions Strategies for effective communication Coping skills	How Hispanic cultural values influence the experience of anger (e.g., Likeability can make the appropriate expression or adaptation of emotions difficult for some Hispanic men)
7) Partnering with my health system/providers	Applying communication skills with healthcare providers Application of self-advocacy strategies in the healthcare setting Components and barriers to assertive communication	How Hispanic cultural values influence interpersonal styles (e.g., respect and likeability can make effective communication between people difficult, but especially between figures of authority such as a doctor and patient). Incorporating the value of Respect into assertive communication components (e.g., Sending an Assertive Message *with Respect* (for example: “With all due respect, I feel/believe that….”)
8) Social connections	Building a social support network Benefits and obstacles to maintaining strong social support Support groups for people like me	Acknowledging the importance of family systems. Examples of local and national support resources for Hispanic SMM living with HIV and Cancer (e.g. *High Impacto*).
9) Healthy behaviors	Healthy lifestyle behaviors (e.g., exercise, diet, smoking) Coping with information overload	Examples of national resources available for Hispanics
10) Wrap up and Program Summary	Plan for continuing work toward goals Additional resources	Review of Hispanic cultural values Examples of national resources available for Hispanic SMM

Given the significant diversity in nationality and acculturation among US Hispanic SMM, we chose to keep the content in English for this pilot study. The content was subsequently reviewed by key stakeholders, including experienced researchers in CBSM content development and adaptation, along with therapists familiar with delivering standard CBSM. We conducted in-house usability testing of the web-based platform to identify and resolve any technical issues. In addition, several training sessions were organized to familiarize intervention facilitators with the new content. These facilitators were master-level Hispanic therapists experienced in working with SMM, cancer survivors, and people living with HIV.

#### Stage 3: Preliminary Adaptation Test

A mixed-methods design was employed following the cultural adaptation of the CBSM intervention. A single-group design with pre- and post-measures was used to assess the feasibility, acceptability, and intended effects of the culturally adapted web-based CBSM intervention. In addition, qualitative data was gathered to evaluate the intervention’s relevance and identify challenges or factors that influenced participation.

### Quantitative Study

#### Participants and Settings

Eligible participants were adults aged 18 or older, fluent in English, diagnosed with HIV and at least one form of solid tumor or blood cancer, at least 30 days post-completion of active primary treatment, self-identified as a sexual minority, cisgender men, and Hispanic, and had reliable access to a device with internet capability. Participants were excluded if they had one of the following exclusionary cancer types: nonmelanoma skin cancer only, brain cancer, eye cancer, or a history of pediatric cancer without a cancer diagnosis as an adult; were currently undergoing primary treatment for their cancer; had inpatient treatment for severe mental illness in the past 12 months, overt signs of severe mental illness, or moderate or higher risk of suicidality at the time of screening; appeared actively intoxicated or otherwise unable to provide full informed consent; or had any medical conditions resulting in a predicted life expectancy of less than 12 months per participant self-report.

Participants were recruited remotely, either online or by phone. We employed several recruitment strategies, including (1) using the University of Miami’s Consent to Contact Database, (2) advertising the study on online recruitment platforms (e.g., ResearchMatch, UMiamiHealthResearch), (3) attending community-hosted events at LGBTQ and HIV organizations to engage with community members, (4) offering monetary incentives for referrals from enrolled study participants, (5) running ads on social networking sites for sexual minorities, and (6) visiting clinics that provide care to people living with HIV to speak with case managers. Potential participants were screened by phone to confirm their eligibility for the study.

#### Procedures

This study received approval from the University of Miami Institutional Review Board. Written informed consent was obtained from all participants prior to data collection. At enrollment, participants completed a psychosocial survey in REDCap that included demographics, medical history, intervention outcomes, and psychosocial factors. They were then enrolled on the SmartManage website to participate in a structured 10-week intervention delivered in a group setting with up to six participants. Weekly group sessions, led by the study research coordinator experienced in conducting CBSM interventions, were held via videoconference and lasted about 90 min. Additionally, participants were requested to complete worksheets through the web-based platform and engage in weekly home exercises. Upon completing the intervention, participants were prompted to fill out the post-intervention questionnaire in REDCap.

#### Measures

##### Feasibility

Feasibility was assessed through enrollment, attendance, and retention rates. The enrollment rate was defined as the proportion of men contacted who agreed to participate. We set a target sample size of 30. Additionally, the intervention was considered feasible if 70% of participants attended at least 70% of all intervention sessions, and 85% of enrolled participants were retained throughout the study, based on benchmarks from previous successful CBSM trials.

##### Acceptability

We assessed the acceptability of the intervention using a post-intervention questionnaire that evaluated the program’s quality, relevance, and ease of use and comprehension.

##### Sociodemographic and Medical Characteristics

Participants self-reported their age, gender, race, marital status, education, employment status, income, nativity, and years of residence in the U.S. Acculturation was assessed using the Bidimensional Acculturation Scale for Hispanics [31]. This 24-item questionnaire produces two subscales: Hispanic and Non-Hispanic and includes responses that range from 1 (almost never) to 4 (almost always). A mean score for each subscale is calculated. Higher scores reflect greater acculturation to the respective cultural domain (Cronbach’s alpha [α’s]: 0.90–0.96.90.96). In addition, a mean score of >2.5 for both the Hispanic and the non-Hispanic subscales are indicative of biculturalism. The medical history questionnaire included cancer diagnosis, treatments received, the most recent CD4 count, HIV viral load status, adherence to anti-HIV medication, and barriers to receiving or continuing HIV care.

##### Intervention Outcomes

Two measures captured HRQoL: Functional Assessment of Cancer Therapy Scale–General (FACT-G) and Medical Outcomes Study HIV Survey (MOS-HIV) [32, 33]. The FACT-G is a 27-item questionnaire that assesses four dimensions of HRQoL (i.e., physical, social, emotional, and functional well-being). The FACT-G total score is computed as the sum of the four subscale scores and has a possible range of 0–108. This measure has been widely used in diverse populations of cancer survivors, including Hispanics, to assess HRQoL (α = 0.88) [34]. The MOS-HIV questionnaire includes 35 items. It assesses 10 health dimensions (i.e., health perceptions, pain, physical functioning, role functioning, social functioning, cognitive functioning, mental health, energy, health distress, and quality of life) [33]. Subscales are scored on a 0–100 scale, with a higher score indicating better health. General stress was measured using the Perceived Stress Scale-14 [35], which is a validated 14-item questionnaire that is widely used and has good psychometric properties (α = 0.84) [35]. Disease-related distress was assessed with the Impact of Event Scale-Revised (IES-R) [36]. This 22-item questionnaire evaluates intrusion, avoidance, and hyperarousal symptoms on a 5-point Likert scale and has demonstrated strong psychometric properties among cancer survivors (subscales α‘s >0.80) [37]. A total IES-R score was created by summing the scores of the three subscales. A total score between 24 and 32 suggests subthreshold PTSD symptoms, whereas a score of ≥ 33 represents a threshold for probable PTSD diagnosis [38].

##### Psychosocial Factors

Participants also completed the Coping Self-Efficacy Scale, a 26-item measure of their confidence in coping with life challenges (α = 0.95) [39]. This instrument utilizes an 11-point scale ranging from 0 (“cannot do at all”) to 10 (“certain can do”) and produces three subscales: problem-focused coping, stopping unpleasant thoughts, and obtaining support from friends and family. Higher scores indicate greater perceived self-efficacy in coping with stress. We assessed perceived social support using the Interpersonal Support Evaluation List (ISEL) [40]. This 40-item questionnaire evaluates four types of support: tangible, appraisal, self-esteem, and belonging, and has been validated among Hispanic cancer survivors. The subscales are summed to determine an overall score, with higher scores signifying higher perceived support. The Everyday Discrimination Scale (EDS) was employed to evaluate daily discrimination experiences among minority populations [41]. This scale consists of nine items, each rated on a six-point scale from 0 (never) to 5 (almost every day). The responses are summed to yield a total score ranging from 0 to 45, with higher scores indicating more frequent experiences of discrimination. The Communication Assessment Tool (CAT) was utilized to assess physicians’ interpersonal and communication skills [42]. This 14-item instrument employs a five-point Likert scale (5 = excellent). A total score is calculated by averaging the responses.

#### Data Analysis

Demographic and disease characteristics were summarized using descriptive statistics. Counts and percentages summarized the distribution of categorical variables, while median, range, mean, and standard deviation were employed for continuous variables. Based on the data distribution, we applied either t-test or the Wilcoxon signed-rank test for paired samples to examine whether the intervention outcomes and psychosocial factors significantly improved after the intervention, utilizing total scores and subdomain scores. In addition, we estimated the effect sizes (Hedges’ *g*) using the mean difference from baseline to post-intervention. Hedges’ *g* incorporates a correction factor to provide a more accurate estimate of the effect size, particularly when samples are small [43]. Values of 0.20, 0.50, and 0.80 are considered to be indicative of small, medium, and large effects [44]. A *p-*value of < 0.05 was deemed significant. Statistical analysis was conducted using SAS software (version 9.4).

### Qualitative Study

#### Sample

All participants were invited to engage in a brief one-on-one final interview to discuss the challenges or factors that influenced their participation in the program, the impact of the program on their health, and suggestions for improvement. Out of the 15 participants, 73% (*n* = 11) agreed to take part in the interview.

#### Procedures

The interviews were conducted via videoconference and facilitated by a postdoctoral trainee experienced in qualitative data collection and analysis. An interview guide was created to explore the challenges and factors influencing participants’ engagement in the program, their impact on health, and suggestions for improvement (Supplement 1). On average, interviews lasted about 20 min and were recorded either in video or audio format. The recordings were subsequently transcribed by members of the study team.

#### Data Analysis

We conducted a rapid qualitative analysis (RQA) to summarize our findings [45]. The advantage of using RQA is that it allows for timely and efficient analysis of qualitative data, enabling quicker decision-making and adaptation of interventions [46]. First, we developed a coding matrix. Then, we summarized responses from each participant and compiled a comprehensive final matrix that condenses data from all interviews. This matrix facilitates data visualization, making it easier to identify patterns and themes. Two co-authors with prior RQA experience reviewed this matrix, drew preliminary conclusions, and presented them to the study team for discussion.

## Results

### Quantitative Findings

#### Feasibility

**Fig. 1:**
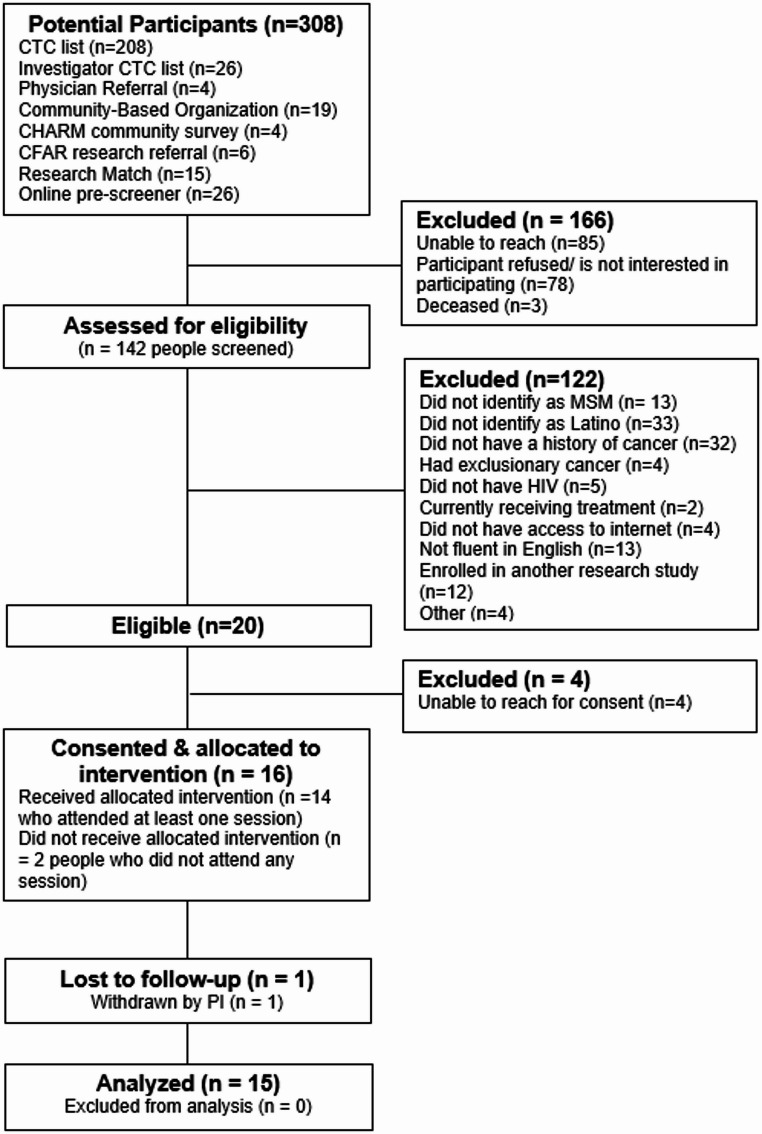
Participant flow diagram. CTC: consent to contact; CHARM: center for HIV and research in mental health; CFAR: center for AIDS research

Our research team contacted 308 potential participants over a 24-month period (Fig. 1). Of these, 28% did not respond to our calls, 25% declined screening, and 40% were ineligible (due to not self-identifying as Latino (27%), not having cancer (26%), not self-identifying as a sexual minority (11%), not being fluent in English (11%), or other reasons (25%)). Of the 20 eligible men, 80% (*n* = 16 participants) signed an informed consent form, but the PI removed one from the study for providing false information. Therefore, 15 Hispanic SMM living with HIV and cancer participated in one of three groups of the 10-week CBSM intervention (overall enrollment rate = 6.8%; 50% target enrollment achieved). On average, participants attended seven out of ten sessions. A total of 73% (11 of 15) attended seven sessions or more. All participants completed both pre- and post-intervention assessments.

Table 2 summarizes the demographic and medical characteristics of the participants. Participants had an average age of 51.2 years (SD: 11.4), ranging from 32 to 62 years. The majority self-identified as gay (80%), while 13% identified as bisexual and 7% as pansexual. Most participants self-identified as White (67%). Regarding education, 53% held a college degree, 27% possessed a master’s or professional degree, and 20% completed high school education. The average annual income was $62,906, ranging from $11,316 to $275,000. Most participants were unemployed (60%), while about a third reported working full-time or part-time (33%). In terms of medical characteristics, one third of the participants were diagnosed with Kaposi’s sarcoma, and another third with gastrointestinal cancer (i.e., stomach, colorectal, or anal cancer). Regarding acculturation, participants reported high identification with both Hispanic and non-Hispanic cultures, with mean scores of 3.06 (SD: 0.4) and 3.2 (SD: 0.6) on the respective subscales. A significant majority (87%) reported biculturalism, with scores above 2.5 in both cultural domains. Furthermore, over 90% of participants reported being highly proficient in Spanish, indicating they speak, read, and write the language well or very well.

**Table 2:** Demographic and medical characteristics

	Mean or frequency	SD or percentage
Age	51.2	11.4
Sex at birth (Male)	15	100
Gender identity (Male)	15	100
*Sexual orientation*		
Gay	12	80
Bisexual	2	13.3
Pansexual	1	6.7
*Race*		
Black	1	6.7
White	10	66.7
Other	2	13.3
Missing	2	13.3
*Acculturation (BAS)*		
Hispanic	3.6	0.4
Non-Hispanic	3.2	0.6
*Education*		
Up to high school	3	20
Some college/College degree	8	53.3
Master’s or Professional degree	4	26.7
Annual Income	$ 62.9 K	$ 76.9 K
*Employment*		
Full-time/Part-Time	5	33.3
Unemployed with disability	5	33.3
Unemployed without disability	4	26.7
Missing	1	6.7
*Cancer type*		
Kaposi’s sarcoma	5	33.3
Gastrointestinal (i.e., Stomach, Colorectal, Anal)	5	33.3
Hematologic (i.e., Lymphoma)	2	13.3
Melanoma	1	6.7
Prostate	1	6.7
Renal	1	6.7

BAS: Bidimensional acculturation scale for Hispanics

#### Acceptability

More than half of the participants (9 out of 15) agreed or strongly agreed that the program was relevant to their health needs. Additionally, 87% (13 out of 15) of participants found the *SmartManage* website easy to navigate and its content easy to understand. The program’s quality was rated as very good or excellent by 80% (12 out of 15) of participants. Furthermore, 93% (14 out of 15) of participants believed the program was helpful for gay and bisexual men living with HIV and cancer. The format of the program was considered effective by 80% (12 out of 15) of the participants, and 93% (14 out of 15) would recommend this program to a friend living with HIV and cancer.

#### Intervention Outcomes

At baseline, participants reported low health-related quality of life, with a mean total FACT-G score below the cutoff score of 70, and low social well-being, with a mean score below the cutoff score of 19 (Table 3). After completing the intervention, participants showed significant improvements in emotional well-being (Mean change = 3.5, t = 3.32, *p* = 0.01). This change corresponded to a large effect size (*g* = 0.79) based on Hedges’ *g*. There were also reductions in pain and health distress scores, although these changes were not statistically significant. In addition, participants reported high-stress levels at baseline, with a mean total score above the cutoff score of 27. While stress levels decreased after the intervention, this change was not statistically significant. Hedges’ *g* confirmed a small effect size in the expected beneficial direction (*g*=−0.15). Based on the IES-R questionnaire, participants also exhibited cancer-related PTSD symptoms, with a mean score exceeding the cutoff score of 24. After the intervention, participants experienced a reduction in PTSD symptoms (Mean change=−10.2, t=−2.06, *p* = 0.06), although this change was not statistically significant. However, they showed a significant reduction in the avoidance symptoms subscale (Mean change=−5.0, S=−2.49, *p* = 0.03). Based on Hedges’ *g*, the intervention achieved medium beneficial effect sizes across PTSD symptom domains and the IES-R total score.

**Table 3:** Means and standard deviations of intervention outcomes and psychosocial factors

	Pre-intervention		Change (Post-Pre)				Hedge’s *g*
	Mean	SD	Mean	SD	Test statistic^§^	*p*	
**Health-related quality of life (FACT-G)**							
Physical well-being	20.3	6.5	−1.5	8	−0.66	0.52^a^	−0.16
Social well-being	16.1	6.8	0.2	4.3	0.19	0.85^a^	0.05
Emotional well-being	15.5	4.2	3.5	3.8	3.32	**0.01** ^a^	0.79
Functional well-being	16.9	6.4	0.4	3.9	0.36	0.73^a^	0.09
Total score	68.9	15.7	2.7	12.1	0.80	0.44^a^	0.19
**Medical outcome study HIV health survey**							
Pain	8.5	2.3	−1	2.4	−1.51	0.16^a^	−0.36
Physical functioning	13.7	3.7	−0.2	4.7	−0.18	0.86^a^	−0.04
Social functioning	4.4	1.7	−0.2	2.5	−0.34	0.74^a^	−0.08
Role functioning	2.7	1.1	0.6	0.8	2.55	**0.03** ^a^	0.61
Mental health	19.9	3.9	0.7	6.3	0.40	0.70^a^	0.09
Health distress	17.3	5.0	−2.5	8.5	−1.01	0.33^a^	−0.24
Cognitive functioning	17.9	5.2	−1.3	6.3	−0.75	0.46^a^	−0.18
Vitality/energy	13.8	3.6	−0.8	3.8	−0.68	0.51^a^	−0.16
General health	14.4	5.4	0.8	5	0.55	0.59^a^	0.13
Health perception	3.2	1.1	0.3	0.8	5.00	0.31^b^	0.35
Changes in health status	3.6	1.2	0.3	1.8	0.49	0.63^a^	0.12
**Perceived stress scale**	31.7	8.4	−1.8	10	−0.64	0.53^a^	−0.15
**Impact of event scale-revised**							
Intrusion	11.7	7.3	−3.2	6.4	−1.82	0.09^a^	−0.43
Avoidance	12.9	6.7	−5.0	7.2	−2.49	**0.03** ^b^	−0.27
Hyperarousal	8.0	6.0	−1.9	6.2	−1.13	0.28^a^	−0.23
Total score	32.6	18.2	−10.2	17.8	−2.06	0.06^a^	−0.44
**Coping self-efficacy**							
Problem-focused coping	6.6	1.5	0.7	1.8	1.38	0.19^a^	0.33
Stopping unpleasant thoughts	6.2	1.6	0.4	1.9	0.75	0.47^a^	0.18
Getting social support	6.1	2.1	0.5	1.9	1.04	0.32^a^	0.25
**Interpersonal support evaluation list**							
Appraisal support	20.1	5.5	0.2	5.4	0.15	0.88^a^	0.04
Tangible support	18.5	6.6	0.1	2.8	0.10	0.92^a^	0.02
Self-esteem support	18.0	4.6	−0.1	3.7	−0.08	0.94^a^	−0.02
Belonging support	17.9	5.3	0.1	2.4	0.12	0.90^a^	0.03
Total score	73.3	19.3	0.6	13.8	0.16	0.88^a^	0.04
**Communication assessment tool**	4.4	0.8	0.1	0.5	0.56	0.59^a^	0.13
**Everyday discrimination scale**	9.7	12.4	−3.2	0.1	−1.63	0.13^a^	−0.39

^a^Paired t-test; ^b^Wilcoxon signed-rank test

^§^Test Statistics: *t* statistic or *S* signed rank statistic

#### Psychosocial Factors

At baseline, participants exhibited moderate levels of perceived self-efficacy in coping with stress, with mean CSES scores ranging from 6.1 to 6.6. They also reported moderate levels of perceived social support, reflected by a mean ISEL total score of 73.3. In addition, participants had a positive perception of physician communication, with a mean CAT score of 4.4, and a low perception of discrimination related to their sexual orientation (mean EDS score = 9.7). There were no statistically significant changes in any of these psychosocial factors following the intervention.

### Qualitative Findings

Table 4 summarizes our qualitative findings, enhanced by illustrative quotes. Participants were primarily motivated to join the sessions by a desire to connect with others who shared similar experiences and to learn from their collective wisdom. The sense of community and validation from peers were significant motivators, as they felt comfortable sharing in a supportive environment. Additionally, participants chose to participate in the study because it was conducted remotely, and they wanted to improve their health, well-being, and coping mechanisms. However, logistical challenges such as medical appointments, work commitments, and family obligations occasionally hindered attendance.

**Table 4:** Qualitative analysis results

Domain	Summary	Quote
Motivation to participate in group sessions	Meeting others with similar medical conditions Learning from others’ experiences and sharing own experiences Remote participation	“The fact that it’s easy. It’s from home. It’s comfortable, and it’s fast, and it’s reliable… the fact that we get to meet also other people in a safe environment… We were in the comfort of our own homes.” (Participant 55)
Barriers to attending the group sessions	Medical appointments Work commitments Family obligations	“because of my work. So, in reality, it’s nothing that you guys can do. You were very good about following up with…” (Participant 240)
Benefits of attending the group sessions	Learning and improving hope and self-process awareness by listening to others Sense of community	“… listening to other people who are going through the same things. That’s helpful to me.” (Participant 239)
Usefulness of the program	Decreased anxiety symptoms Improved stress management and mood Increased motivation for healthy behaviors (e.g., exercise and healthy eating) Openness to discuss health status with family and friends and ask for help Respect for other points of view and expecting respect from others Helped with organizing medical appointments and records	“The breathing exercises. To me it was phenomenal… I could feel the effects of them. You know, throughout the week, and at a lot of times. And I still, I’m still doing it.” (Participant 240)
Representation of Latino sexual minority living in the US	Relevant and accurate representation Less specific for those with a higher level of acculturation	“Yeah, of course, I related to a lot of the stereotypes of Latinos… other people’s expectations of me… It was all pretty accurate.” (Participant 239)
Relevance of the Latino cultural values	Strong and relevant for the majority Less applicable for those acculturated or without a support system	“Yeah. Because as Latinos, we are private and reserved, and we don’t like to ask for help. And we don’t like to tell people our business so. Yes… It makes you realize that… the illness is not your fault.” (Participant 284)
Suggestions to improve motivation or to improve the program	Offer different session times to accommodate different schedules Include content in Spanish Add more content and allow access after program completion Offering more rewards	“…Most of the people, virtually they’re looking for a stipend.” (Participant 197)

Participants found the sessions beneficial in various ways, including enhancing self-awareness, reducing anxiety, feeling “less alone” through emotional support from the group, and fostering a sense of community. They also found the program useful for tracking symptoms, organizing medical records, and providing valuable information and practical skills, such as meditation exercises, stress management techniques, and coping strategies. The content was generally perceived as culturally relevant and accurately representing Hispanic SMM; however, some participants suggested a more nuanced approach to acknowledge the diverse experiences within the Hispanic community. Additionally, the program’s focus on Hispanic cultural values was typically well-received and considered relevant to the participants’ lives.

Suggestions for improvement included shortening session durations, increasing scheduling flexibility (e.g., various time slots), incorporating Spanish content, providing additional opportunities for social interaction, offering more incentives to boost attendance, enhancing the layout of the web platform, following up after program completion, and adding more challenging exercises.

## Discussion

Although advancements in HIV treatment have significantly extended the lifespan of individuals living with the virus, Hispanic SMM with HIV and cancer continue to face considerable challenges. Stigma, bias, and unforeseen health issues, such as an increased risk of cancer, contribute to poorer HRQoL, lower cancer survival rates, and heightened mental health issues like depression and anxiety. However, very few programs specifically address the unique needs of Hispanic SMM living with HIV and cancer. To bridge this gap, we culturally adapted a CBSM intervention to alleviate the disproportionate symptom burden and enhance HRQoL among this vulnerable and underrepresented population.

Despite dedicating significant time and effort as well as utilizing various recruitment strategies over a 24-month period, we successfully recruited only 50% of our target sample size. This low recruitment rate was likely due to our strict eligibility criteria (i.e., adult men who self-identify as sexual minorities, are Hispanic, and are living with both HIV and cancer). Additional factors such as fear of discrimination or judgment, competing priorities, distrust of the medical system, concerns about confidentiality, and low awareness of the need for treatment may also have contributed to the recruitment challenges [47, 48]. Future research seeking to recruit similar populations should consider a multi-faceted approach, including fostering trust and partnerships with community organizations that serve Hispanic LGBTQ + individuals affected by HIV and cancer, raising awareness about clinical trials and their significance within these communities, providing the study in Spanish, and potentially offering more substantial incentives to compensate for time and participation.

While recruitment proved challenging, other feasibility metrics were positive. Participants attended an average of seven out of ten sessions, demonstrating a high level of engagement with a 73% attendance rate for seven or more sessions. This level of engagement is particularly noteworthy considering the complex challenges faced by this population, including managing multiple health conditions and psychosocial stressors. Furthermore, all participants completed pre- and post-intervention assessments, indicating a willingness to contribute to the study despite these challenges. Participants also reported high levels of acceptability, which aligns with prior web-based CBSM studies in other cancer subpopulations [49]. Most participants in our study found the program relevant to their health needs and had positive perceptions of its quality. The strong belief in the program’s helpfulness for Hispanic SMM living with HIV and cancer, along with a high recommendation rate, underscores the program’s perceived value and relevance. Moreover, 87% of participants found the SmartManage website easy to navigate and its content easy to understand, which is crucial for the successful implementation of any technology-based intervention. This suggests that the program’s design was user-friendly and accessible, minimizing potential barriers to engagement.

Baseline data reveal significant challenges faced by Hispanic SMM living with HIV and cancer in our study. Their low mean scores on the FACT-G and social well-being subscale indicate diminished HRQOL and social support, consistent with the complex interplay of factors affecting this population, such as managing multiple chronic conditions, navigating social stigma, and potentially encountering discrimination. Elevated baseline stress levels and the presence of cancer-related PTSD symptoms, evidenced by high IES-R scores, further underscore the substantial burden experienced by this group. These findings suggest that the cancer experience, beyond its physical symptoms, notably impacts psychological health, contributing to PTSD symptomatology. Collectively, this baseline data highlights the critical need for targeted interventions. Although recruitment in this population will remain challenging, these obstacles should not prevent high-quality intervention research, as shown by the high prevalence of psychological distress and poor HRQOL among this population.

Despite our small sample size, our intervention showed promise, yielding statistically significant improvements in emotional well-being and the PTSD avoidance subscale. Participants also experienced trends toward reductions in overall PTSD symptoms, pain, health distress, and stress levels. Qualitative data from interviews further revealed participant-reported benefits from attending the group sessions, such as reduced anxiety, a decreased sense of isolation due to emotional support from the group, and the development of a sense of community. These promising preliminary findings support the refinement of the intervention. In line with the fourth stage in the Barrera and Castro heuristic framework, we will refine our intervention by translating all the content into Spanish, offering group sessions in Spanish, increasing scheduling flexibility, adding more exercises that provide opportunities for discussion and social interaction, and including additional practical resources (e.g., meditation exercises and stress management strategies).

The limitations of the study include a small sample size, the pre-post design, and the absence of a comparison condition. Therefore, following the intervention refinements, future efforts will involve testing the improved version of this CBSM intervention in a randomized controlled trial with a larger sample size to ensure the study is adequately powered to detect statistically significant intervention effects.

## Conclusions

Our findings highlight the significant burden of psychological distress and poor HRQOL faced by Hispanic SMM living with HIV and cancer, underscoring the need for targeted interventions. The high acceptability and engagement with our intervention, combined with promising preliminary results, indicate that a culturally adapted CBSM intervention is both relevant and beneficial to this underserved population. However, recruitment challenges stemming from stringent eligibility criteria and other obstacles stress the necessity for more inclusive and flexible recruitment strategies in future research.

## Supplementary Information

Below is the link to the electronic supplementary material.

Supplementary material 1 (DOCX 19.8 kb)
