# Supplementary material for: Cultural Adaptation of a Web-Based Cognitive Behavioral Stress and Self-Management Intervention for Hispanic Sexual Minority Men Living with HIV and Cancer: A Mixed-Methods Feasibility Study
Source: AIDS Behav. 2025 Oct 28;30(3):928–41. doi: 10.1007/s10461-025-04945-y (PMC12989017; doi:10.1007/s10461-025-04945-y)
Supplement: Supplementary file 1 — Supplementary material 1 (DOCX 19.8 kb) [file 10461_2025_4945_MOESM1_ESM.docx]

**Final Interview Guide**

**Pre-Interview Checklist:**

___ Review participant’s attendance

___ Confirm whether participant consented to video- or audio-recording.

**Introduction:**

Thank you for participating in this interview. The purpose of our conversation is to understand the factors affecting your participation in the program, the impact of the program on your health, and suggestions for improvement. We want to use this information to make better strategies for engaging patients in supportive programs.

The interview will last approximately 10-20 minutes and it will be audio or video-recorded and transcribed to review at a later time. If you decline audio or video-recording, you will not be penalized in any way and extensive notes will be taken to capture everything you say. You have the right to stop this interview at any time if you feel like doing so or you can skip any question that you do not want to answer for any reason. Feel free to ask if any of our questions are not clear to you. Please give as much information as you can. During the interview, we will use first names, but the comments in the final reports will not be linked to any specific names. Your responses will remain anonymous. After our discussion, you will receive $25 for completing the final interview.

Are there any questions before we begin?

1. **Attendance:**

- For those who participated in at least 1 session: What motivated you to participate in the group sessions?
- What (if anything) got in the way of you attending the group sessions?
- What might motivate you to attend group sessions like these in the future?

1. **Impact:**

- For those who attended <5 sessions: What benefits did you expect from participating in the group sessions?
- How useful did you find the group sessions?
- How (if at all) did the program lead to changes in
  - Your health (e.g., physical, and psychological symptoms such as your mood, your stress levels)?
  - Your interaction with your caregivers, family members, and friends?
  - Your interaction with your healthcare providers?
- How useful did you find the information presented on the website?
- How useful were the exercises?
- How much did you relate to the material presented as a Latino sexual minority men?
  - Probe: Do you feel it captures your experience as a Latino SMM living in the US?
- How relevant did you find the Latino cultural values presented in the program?

1. **Suggestions for improvement:**

- What (if anything) could we improve to help keep you motivated to use the program?
- What suggestions do you have for improving the program?

Are there any other experiences or comments you would like to share that you feel have not been covered in this interview?

**Closing Checklist**:

___ Thank the participant for his time.

___ Remind him that all responses will remain confidential.

___ Send compensation.
